# Rhino-orbito-cerebral mucormycosis infection in a 4-year-old Egyptian girl

**DOI:** 10.1016/j.mmcr.2022.07.002

**Published:** 2022-07-09

**Authors:** Ahmed Sorour, Amani Said Abdelrahman, Amir Abdelkareem, Ahmed Kadry, Ahmed Gamal

**Affiliations:** a Department of Plastic Surgery, Al-Matria Teaching Hospital, Cairo, Egypt; b Department of Plastic Surgery, El-Sahel Teaching Hospital, El Sahel, Cairo, Egypt; c Department of Dermatology and Venerology, Al-Azhar University, Cairo, Egypt; d Department of Dermatology, Case Western Reserve University, Cleveland, OH, USA; e Department of Dermatology, School of Medicine, October 6 University, Giza, Egypt

**Keywords:** Mucormycosis, Children, Pediatric, Rhino-orbito-cerebral

## Abstract

Rhino-orbital cerebral mucormycosis is a rare, potentially fatal fungal infection that usually affects diabetic and immunocompromised adults. Recently, an increase in the frequency of this infection in children has been reported, especially in neutropenic patients and premature babies. In this article, we are reporting a case of rhino-orbital cerebral mucormycosis in a 4-year-old girl with a newly diagnosed diabetes mellitus. Tissue biopsy, fungal culture, and imaging were used to confirm the diagnosis. The infection was successfully treated with combined surgical debridement and antifungals.

## 1 Introduction

Mucormycosis is an uncommon fungal infection that particularly affects immune-compromised and diabetic patients [1]. Several genera of this fungus were isolated from infected humans, such as *Rhizopus* and *Mucor*, which are characterized by the broad, irregularly branched, non-septate hyphae. Although this infection is more frequently reported in adults, recently, several cases among the pediatric population have been reported in the literature [2,3].

Mucormycosis may have different presentations depending on the site of infection. Additionally, the incidence of each type is strongly linked to the underlying risk factor and age. For example, the rhino-orbito-cerebral type is reported to be more common among patients with uncontrolled diabetes, especially in adults [4,5]. While the gastrointestinal type occurs more frequently in children and young adults [6,7]. Furthermore, the cutaneous type is reported to be more common in healthcare settings, as skin breaks facilitate the fungus penetration into the tissue, such as at the intravenous catheter and insulin injection sites [8].

Given the nature of this fungus and its ability to rapidly invade the vascular tissue causing massive necrosis, early detection as well as rapid aggressive intervention, is critical and may aid in the reduction of the high mortality rate associated with this infection [9]. Here, we are reporting a case of Rhino-orbital-cerebral mucormycosis (ROCM) infection in a 4-year-old girl at Al-Matria Teaching Hospital, Cairo, Egypt.

## 2 Case

A 4-year-old white girl first presented to the pediatric emergency room with a disturbed conscious level and a history of nausea, vomiting, polyuria, polydipsia, headache, and abdominal pain that persisted for 7 days duration. Diagnosis of untreated diabetic ketoacidosis (DKA) was confirmed with a blood glucose level of 600 mg/dl and the presence of ketone bodies in the urine. Furthermore, arterial blood gas analysis revealed a pH of 6.8, partial pressure of carbon dioxide of 10 kPa, and serum bicarbonate of 3 mEq/L. The patient was admitted to the hospital and received a 10 ml/kilogram bolus of normal saline over 30 minutes. Next, maintenance fluid was calculated using the Holliday-Segar formula (10 kg [patient’s weight] × 100 ml per day). Potassium level was corrected by administration of potassium chloride in normal saline (0.9%) at a concentration of 40 mmol/500 ml. One hour later, insulin infusion was started at a rate of 0.05 units/kilogram/hour using a syringe pump. The fluid maintenance regimen was adjusted based on the serum glucose and potassium levels.

**Fig. 1:**
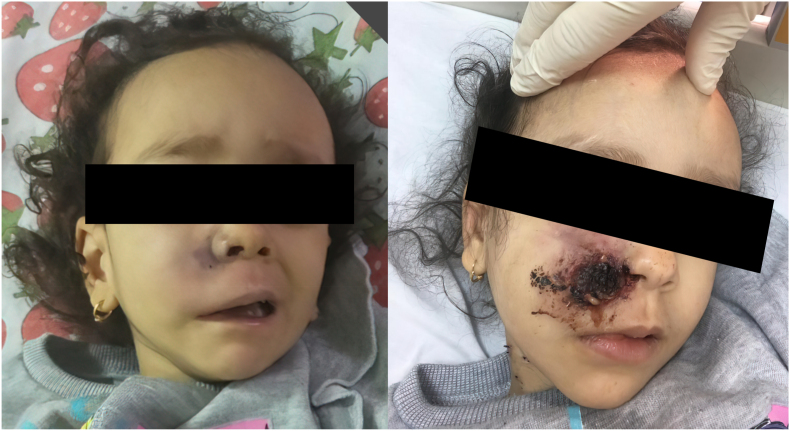
Photographic representation of the bluish discoloration that was noted on day +1 with subsequent progression into tissue necrosis over the next few days.

Physical examination on day +1 showed newly developed ptosis and swelling of the right upper eyelid. Additionally, bluish discoloration of the skin over the right maxilla and swelling that extended to the right angle of the mouth inferiorly were noted (Fig. 1). The patient’s mother denied any recent history of trauma to the face. Further examination of the oral cavity revealed dark bluish discoloration of the soft and hard palate surrounded by inflamed mucosa. Primary periorbital cellulitis secondary to bacterial infection was suspected and empiric antibiotic treatment was initiated at day +1.

**Fig. 2:**
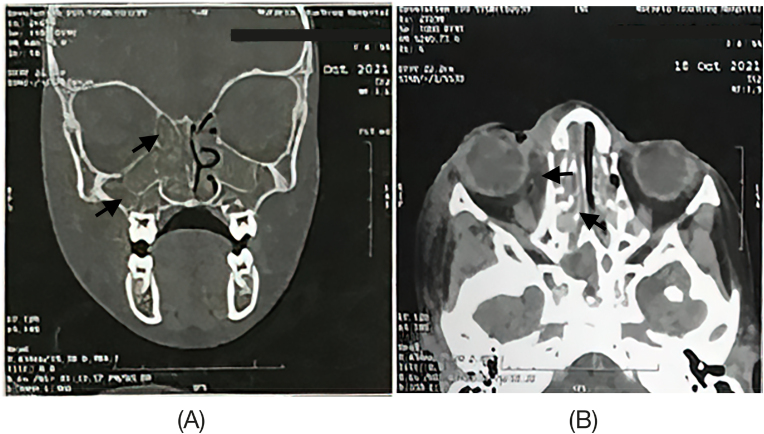
Non-contrast CT scans of the paranasal sinuses and orbit on day +2. (A) Coronal view showing complete obliteration of the left and right maxillary and ethmoidal sinuses by mucosal thickening and retained secretions with obliterated osteomeatal units. (B) Axial view showing right preseptal and peri-orbital soft tissue swelling.

Facial computed tomography (CT) was done on day +2 which revealed pan-sinusitis and near-total obliteration of the left maxillary and left ethmoidal nasal sinuses with total obliteration of the rest of the paranasal sinuses by mucosal thickening and retained secretions. This was also associated with right orbital and periorbital cellulitis (Fig. 2).

**Fig. 3:**
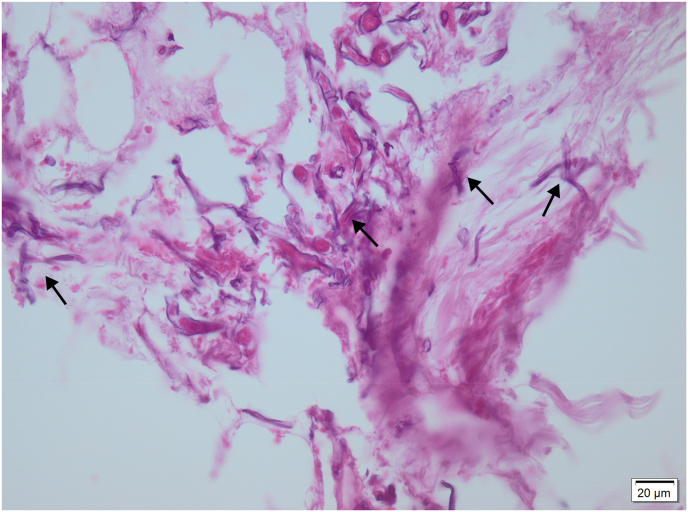
Histopathology of Hematoxylin-eosin-stained section showing non-septate fungal hyphae.

Later, nasosinusal endoscopic exploration was done on day +3 which revealed inflamed nasal and maxillary sinus mucosa. Pathological evaluation of tissue biopsy revealed large non-septate branching hyphae, infiltrating devitalized bone spicules, and spores at a background of acute and chronic inflammatory cells which confirmed the presence of fungal infection at day +4 (Fig. 3). The diagnosis was later confirmed with a fungal culture based on morphology. The antibiotics were discontinued, and empiric antifungal treatment was initiated at day +4 in the form of liposomal amphotericin B 1 mg per kilogram every 24 hours. On day +5, the discolored areas turned black and became necrotic (Fig. 1).

A second CT on day +8 revealed bone erosion in the medial wall of the maxillary sinus, hard palate, and superior and inferior turbinate. Furthermore, Magnetic resonance imaging (MRI) revealed pan-sinusitis as well as right mastoiditis, orbital cellulitis, cavernous sinus, and internal carotid artery thrombosis for which the patient was treated with subcutaneous enoxaparin sodium 2 mg per kilogram every 24 hours.

**Fig. 4:**
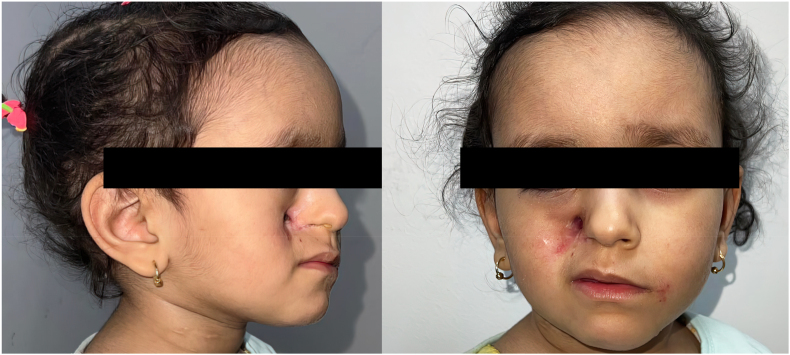
Photographic representation of the patient’s condition on follow-up. Deformation of the face over the maxillary sinus can be noted on the right side compared to the left side.

Surgical intervention was performed on day +19 to remove the necrotic tissue and reduce the risk for further progression. Additionally, the liposomal amphotericin B dose was increased to 5 mg per kilogram every 24 hours and combined with the application of a gauze soaked with the same antifungal within the sinus cavity. This regimen was continued for a total of 8 weeks. Gradual improvement of the patient’s condition was noticed on day +29. After completion of the amphotericin regimen, the patient was discharged on day +86 following confirmation of resolution of infection with negative cultures and imaging. Follow-up visits were scheduled every week with the planning for surgical reconstruction of the facial deformation that resulted from the tissue debridement (Fig. 4).

## 3 Discussion

Based on epidemiological data, mucormycosis infection is very rare among the pediatric population. Most studies report an average age of presentation around 40 to 50-year-old. [4] However, the incidence of infection has been increasing over the past years. [10] This was demonstrated by the data provided by Pana et al. that included 63 pediatric cases of mucormycosis, with an average age of 13-year-old, reported from 15 countries between 2005 and 2014. Interestingly, in 52.3% of the cases, the infection was associated with malignancies compared to 4.8% with DM. [10]

We presented a case of ROCM in a 4-year-old diabetic child. Several studies have shown a strong association between diabetes mellitus, especially in uncontrolled patients, and mucormycosis. [7] In fact, in a recent epidemiologic study that was conducted in the Middle East, 49.7% of the patients (aged between 23 and 59 years old) diagnosed with mucormycosis infection were diabetic. [8] However, in the pediatric population, the incidence-risk factor association was shown to be different compared to adults. In a review that included 157 cases of zygomycosis in pediatric patients, neutropenia (18%), prematurity (17%), and malignancy (16%) were the most common underlying risk factors followed by DM accounting for 15% of the cases [2,3]. Furthermore, in older children, cutaneous and gastrointestinal types were the most common forms of infection followed by ROCM. While in neonates, the gastrointestinal type was more common with a high mortality rate.

Generally, *R. arrhizus* is the most commonly isolated species worldwide. [7] In our case, the performance of any identification process was not possible financially and we depended on histopathologic analysis, culture, as well as clinical presentation to establish the diagnosis.

Infection with this organism can be acquired by inhalation of the fungal spores that reside in the soil with subsequent germination and angioinvasion that occur in susceptible patients. This in turn results in massive necrosis and the spread of infection to the surrounding tissue accounting for the severe morbidity and high mortality reported in these patients. [7,9]

Initially, in ROCM, the infection affects the nasal turbinates resulting in fever, headache, and nasal discharge. Then, the condition may progress into pan-sinusitis and local tissue necrosis affecting the nasal mucosa, palate, overlying facial skin, orbit, and brain. [9,11] Furthermore, thrombosis of the surrounding blood vessels and venous sinuses may occur, thus aggravating tissue necrosis and increasing the mortality risk. In our case, the child was complicated with cavernous sinus and internal carotid artery thrombosis that necessitated the administration of an anticoagulant. This in turn resulted in several brain infarcts with the affection of the patient’s mental status.

We treated the patient with a combined surgical and antifungal approach as it was shown to have a favorable outcome compared to the use of either treatment modalities alone. [10] Furthermore, we used liposomal amphotericin B intralesionally which has been reported to be effective in eliminating the infection while reducing the risk of systemic toxicity. [12] The intralesional application can facilitate the delivery of the antifungal agents to their targets since vascular occlusion may interfere with achieving the appropriate tissue concentration of the systemically administrated drug. Additionally, surgical debridement has a critical role in eliminating the acidotic environment created by the ischemic and necrotic tissue, which is ideal for fungal growth. [13]

Although mucormycosis is rare in children, a high level of suspicion should be practiced when assessing these patients, especially those with associated risk factors. Additionally, early detection and management are critical and may prevent devastating consequences. With increasing incidence in children, more studies are needed to investigate the possible underlying cause along with the development of novel identification and treatment methods that may facilitate early diagnosis and improve the overall survival rate.

## Declaration of competing interest

All authors declare no conflict of interest.
